# Resveratrol Alleviates 27-Hydroxycholesterol-Induced Senescence in Nerve Cells and Affects Zebrafish Locomotor Behavior via Activation of SIRT1-Mediated STAT3 Signaling

**DOI:** 10.1155/2021/6673343

**Published:** 2021-06-21

**Authors:** Jiao Liu, Kailin Jiao, Qian Zhou, Jun Yang, Keke Yang, Chunyan Hu, Ming Zhou, Zhong Li

**Affiliations:** ^1^The Key Laboratory of Modern Toxicology of Ministry of Education, School of Public Health, Nanjing Medical University, No. 818 East Tianyuan Rd., Nanjing 211166, China; ^2^Department of Nutrition, The Affiliated Suzhou Hospital of Nanjing Medical University, No. 16 West Baita Rd., Suzhou 215000, China; ^3^Shenzhen Academy of Metrology & Quality Inspection, Shenzhen 518131, China

## Abstract

The oxysterol 27-hydroxycholesterol (27HC) is the first identified endogenous selective estrogen receptor modulator (SERM), which like endogenous estrogen 17*β*-estradiol (E_2_) induces the proliferation of estrogen receptor- (ER-) positive breast cancer cells *in vitro*. However, 27HC differs from E_2_ in that it shows adverse effects in the nervous system. Our previous study confirmed that 27HC could induce neural senescence by activating phosphorylated signal transducer and activator of transcription, which E_2_ could not. The purpose of the present study is to investigate whether STAT3 acetylation was involved in 27HC-induced neural senescence. Microglia (BV2 cells) and rat pheochromocytoma cells (PC12 cells) were used *in vitro* to explore the effect of resveratrol (REV) on 27HC-induced neural senescence. Senescence-associated *β*-galactosidase (SA-*β*-Gal) staining was performed using an SA-*β*-Gal Staining Kit in cells and zebrafish larvae. Zebrafish were used *in vivo* to assess the effect of 27HC on locomotor behavior and aging. We found that 27HC could induce senescence in neural cells, and REV, which has been employed as a Sirtuin-1 (SIRT1) agonist, could attenuate 27HC-induced senescence by inhibiting STAT3 signaling via SIRT1. Moreover, in the zebrafish model, REV attenuated 27HC-induced locomotor behavior disorder and aging in the spinal cord of zebrafish larvae, which was also associated with the activation of SIRT1-mediated STAT3 signaling. Our findings unveiled a novel mechanism by which REV alleviates 27HC-induced senescence in neural cells and affects zebrafish locomotor behavior by activating SIRT1-mediated STAT3 signaling.

## 1. Introduction

Cholesterol can be oxidized in the body by the side chain to form oxidized cholesterol. The side chains contain hydroxyl groups and play multiple roles in lipid metabolism. Among the cholesterol oxidation products, the most abundant and studied category is 27-hydroxycholesterol (27HC) [1]. The 27HC is found almost entirely in the peripheral circulation and can flow into the brain through the blood-brain barrier (BBB). The 27HC plays an important role in maintaining the balance of brain and extracerebral cholesterol [2]. The 27HC is a selective estrogen receptor modulator (SERM). The 27HC functions like endogenous estrogen 17*β*-estradiol (E_2_) and induces the proliferation of estrogen receptor- (ER-) positive breast cancer cells in vitro [3]. Classically, E_2_ plays a protective role in neurodegenerative diseases. However, it has been reported that 27HC induces adverse effects in the nervous system, distinguishing it from E_2_ [4]. This is consistent with our previous study, which confirmed that 27HC could induce nerve cell senescence as well as tumor invasion and migration but that E_2_ could not [5–7].

Signal transducer and activator of transcription 3 (STAT3) is a member of the STAT family of transcription factors and is involved extensively in cellular functions such as cell proliferation, cell differentiation, immune response, cell survival, and apoptosis [8–11]. Following receptor activation, Tyr705 phosphorylation of STAT3 induces homo- or heterodimerization of phospho-STAT3 (p-STAT3). Dimerized STAT3 translocates to the nucleus and binds to the promoters bearing cognate DNA-binding sequences [12]. In addition, Lys685 residues of STAT3 can be acetylated by p300 and play a decisive role in the transcriptional activity of STAT3, which stabilizes the dimer formation of STAT3 and is required for activating STAT3 transcription [13, 14]. Sirtuin-1 (SIRT1), a class III protein deacetylase, potentially plays a role in diabetes, inflammation, and neurodegeneration [15]. It has been reported that SIRT1 counteracts the activation of acetyl-STAT3 (Lys685) and can induce the loss of viability and increase in senescence in gastric cancer (GC) cells [16]. However, the function of SIRT1 and acetyl-STAT3 (Lys685) in 27HC-induced neural senescence and its mechanism remain unclear.

Resveratrol (REV), a polyphenol found in red grapes, red wine, and other plant foods, is receiving increasing attention owing to its medical potential. A recent report showed that REV could increase SIRT1 expression and reduce the senescence-associated secretory phenotype via the SIRT1/NF-*κ*B pathway in the gut of the fish Nothobranchius guentheri [17]. REV has also shown benefit in neurodegenerative diseases, which are a group of chronic, progressive disorders characterized by the gradual loss of neurons in several areas of the central nervous system (CNS) [18].

BV2 cell lines retain a variety of morphology, characterization, and functional characteristics of microglia [19]. PC12 cell has similar morphological, physiological, and biochemical functions with neurons and is widely used in the study of various neurological diseases [20, 21]. In the present study, using 27HC-treated cells (BV2 and PC12) and zebrafish larvae, we aimed to address the following questions: (1) whether STAT3 acetylation is involved in 27HC-induced neural senescence, (2) whether SIRT1 can regulate STAT3 acetylation by 27HC treatments, (3) whether reactive oxygen species (ROS) can regulate SIRT1 by methylation following 27HC-exposure, and (4) whether REV can improve 27HC-induced adverse effects.

## 2. Materials and Methods

### 2.1. Reagents and Cell Culture

The 27HC (purity ≥ 99.9%) was purchased from Santa Cruz Biotechnology (Santa Cruz, CA, USA) and was dissolved in absolute ethanol at a stock concentration of 20 mM and then stored at -80°C. C646 was purchased from MedChem Express (MCE, USA) and was dissolved in dimethyl sulfoxide (DMSO) at a stock concentration of 10 mM and then stored at -80°C. N-Acetylcysteine (NAC) (an antioxidant) was purchased from Beyotime Co., Ltd. (China). REV was purchased from Xuhuang Biotechnology Co., Ltd. (Xi'an, China). Azacitidine (AZA) and S-adenosyl methionine (SAM) were purchased from Sigma-Aldrich. All other reagents were of the highest analytical grade available. Anti-Ac-STAT3^Lys685^ (2523), anti-SIRT1 (8469), anti-DNMT1 (5032), and anti-p-STAT3^Tyr705^ (9145) were purchased from Cell Signaling Technology (CST, USA).

BV2 microglial cells and PC12 rat pheochromocytoma cells were purchased from the Institute of Basic Medical Sciences of the China Science Academy. The cells were maintained in high glucose Dulbecco's Modified Eagle's Medium (DMEM) supplemented with 10% fetal bovine serum, 100 units/ml penicillin, and 100 mg/ml streptomycin. They were maintained in a humidified 5% CO_2_/95% air environment at 37°C.

### 2.2. Zebrafish Husbandry and Collection of Embryos

AB strain zebrafish (Danio rerio), purchased from Nanjing EzeRinka Biotechnology Co., Ltd., were kept in a recirculating system at 28.5°C, with a 14 h light/10 h dark cycle 7 days per week [22]. The fish system water was aerated and measured daily to maintain a dissolved oxygen concentration of 7.5–8 mg/l and pH of 7.0–7.6. The fish were fed with live brine shrimp (Artemia nauplii, Tianjin Ocean Pal Carol Biotech Co., Tianjin, China) twice per day. Male and female adult fish were transferred in pairs at a ratio of 2 : 1 to a spawning aquarium overnight, and the spawning finished during the first 30 min of the light cycle the next morning. Fertilized eggs were collected, then washed with embryo medium (0.137 M NaCl, 5.4 mM KCl, 0.25 mM Na_2_HPO_4_, 0.44 mM KH_2_PO_4_, 1.3 mM CaCl_2_, 1.0 mM MgSO_4_, and 4.2 mM NaHCO_3_) and incubated in Petri dishes at 28 ± 1°C until methamidophos exposure experiments [23]. All experiments were performed with the approval of the Ethics Committee of Nanjing Medical University. All procedures were performed according to the guidelines of the Animal Care Committee of Nanjing Medical University.

### 2.3. The 27HC Treatment of Zebrafish Embryos and Larvae

Test solutions with 27HC were freshly prepared using embryo medium before exposure experiments. The concentrations of the test solutions were 0 (control), 10 *μ*M, 20 *μ*M, 30 *μ*M, or 40 *μ*M. Zebrafish embryos 36 h postfertilization (hpf) were transferred to the solutions in a 6-well plate with 30 larvae per well, and the plate was incubated at 28 ± 1°C under the same light cycle as the adults throughout the 4 days postfertilization (dpf) exposure period. The exposure solutions were changed daily to ensure nominal concentrations of 27HC and water quality.

### 2.4. Locomotor Behavior Observation

Larvae locomotor activity was monitored at 4 dpf using a Noldus DanioVision (Noldus, Netherlands). Zebrafish embryos 4 dpf were transferred to 96-well plates (one per well). The appropriate amount of test solution per well was used to cover larvae. Following a 5 min habituation period, the locomotor activity was recorded on video for 3 min [24]. Automated analysis of traces from EthoVision XT software (Noldus, Netherlands) was used to assess distance traveled (mm) and mean speed (mm/s) (*n* = 30) [25].

### 2.5. SA-*β*-Gal Assay and Quantitation in Cells and Zebrafish Larvae

Senescence-associated *β*-galactosidase (SA-*β*-Gal) staining was performed using an SA-*β*-Gal Staining Kit (Beyotime Co., Ltd.; C0602) according to a standard protocol [26]. SA-*β*-Gal-positive cells were considered to be cells with a blue or bright blue color by microscopy. And more than 500 cells in 6 random fields were counted to determine the percentage of SA-*β*-Gal-positive cells in the total cells.

Zebrafish larvae were fixed in 4% paraformaldehyde in phosphate-buffered saline (PBS) at 4°C overnight and then washed 3 times for 1 h in PBS-pH 7.4 and for a further 1 h in PBS-pH 6.0 at 4°C. Staining was performed overnight at 37°C in 5 mM potassium ferrocyanide, 2 mM MgCl_2_, and 1 mg/ml X-gal in PBS adjusted to pH 6.0. All larvae were photographed under the same conditions using reflected light under a dissecting microscope. SA-*β*-Gal activity of the neural tube site of zebrafish larvae in each group (*n* = 30) was quantitated using a selection tool in Adobe Photoshop for a color range that was chosen by 25 additive blue color selections of regions that showed visually positive SA-*β*-Gal staining. To quantitatively examine SA-*β*-Gal levels in vivo, we generated high-resolution digital images that enabled us to select stained pixels using Adobe Photoshop analysis software and then to calculate the percentage of stained pixels out of the net total in each case.

### 2.6. Detection of ROS Generation in Larvae

For in vivo ROS detection, live 4-dpf-old embryos were incubated with dichlorofluorescein diacetate (DCFH-DA) at 37°C for 30 min. The fluorescent signal was observed using a fluorescence microscope. To quantify the ROS levels, the DCFH fluorescence intensity was analyzed by a BD FACSCalibur (BD Biosciences, San Jose, CA, United States) at an excitation wavelength of 488 nm and an emission wavelength of 525 nm. The experiments were repeated a minimum of three times.

### 2.7. Western Blot

Total protein was extracted using Radio Immunoprecipitation Assay (RIPA) buffer (Beyotime Co., Ltd.). Then, the protein concentrations were measured with the bicinchoninic acid (BCA) kit (Beyotime Co., Ltd). Afterwards, proteins (20 *μ*g) were separated by 10% sodium dodecyl sulfate-polyacrylamide gel electrophoresis followed by transfer to polyvinylidene fluoride membranes (Millipore, Billerica, USA). After blocking with 10% nonfat milk, membranes were incubated with the primary antibody at 4°C overnight, using antibodies for Ac-STAT3^Lys685^, SIRT1, DNMT1, and p-STAT3^Tyr705^ (CST, 1 : 500 dilution) and *β*-actin (Beyotime Co., Ltd.; 1 : 500 dilution). Then, the membranes were incubated with horseradish peroxidase-conjugated secondary antibodies (Beyotime Co., Ltd.; 1 : 2,000 dilution) for another 1 h at 37°C. The immune complexes were detected using an enhanced chemiluminescence kit (CST). Bands were normalized using *β*-actin to correct for differences in the loading of the proteins. Densitometric analysis was conducted using Image-Pro Plus 6.0 software as described previously [27].

### 2.8. Cell Transfection

BV2 and PC12 cells were seeded in 6-well plates at a density of 1 × 10^5^ per well for 24 h. Then, the cells were transiently transfected with NC-siRNA (25 nM) or SIRT1-siRNA (25 nM) by Lipofectamine 2000 (Invitrogen) for 6 h, respectively, following the manufacturer's instructions [27]. Then, the media of cells were replaced with MEM (10% FBS) without penicillin-streptomycin. NC-siRNA (si-NC) and SIRT1-siRNA (si-SIRT1) were obtained from Santa Cruz Biotechnology (Santa Cruz, CA, USA), and the sequences are listed in Table 1.

### 2.9. Quantitative Real-Time Polymerase Chain Reaction (qRT-PCR)

All primers were synthesized by RiboBio Co. (Guangzhou, China). Total RNA was isolated using TRIzol (Invitrogen, Carlsbad, CA) according to the manufacturer's recommendations. For detection of mRNA, total RNA (2 *μ*g) was transcribed into cDNA using AMV Reverse Transcriptase (Promega, Madison, WI), according to the manufacturer's instructions. Primer sequences are shown in Table 2. The qRT-PCR was performed using the LightCycler 96 SYBR Green I Master Mix (Roche).

### 2.10. Statistical Analysis

Data sets were compared using GraphPad 6.0 (GraphPad Software, Inc., La Jolla, CA, USA). Data are presented as the mean ± standard deviation (SD). The differences between two groups were analyzed using two-tailed Student's *t*-test. For the repeated measure data, a one- or two-way analysis of variance (ANOVA) followed by a Sidak's multiple comparisons test was used. Differences with *P* values < 0.05 were considered statistically significant.

## 3. Results

### 3.1. Effects of Acetylation on 27HC-Induced Senescence in Neural Cells

**Figure 1:**
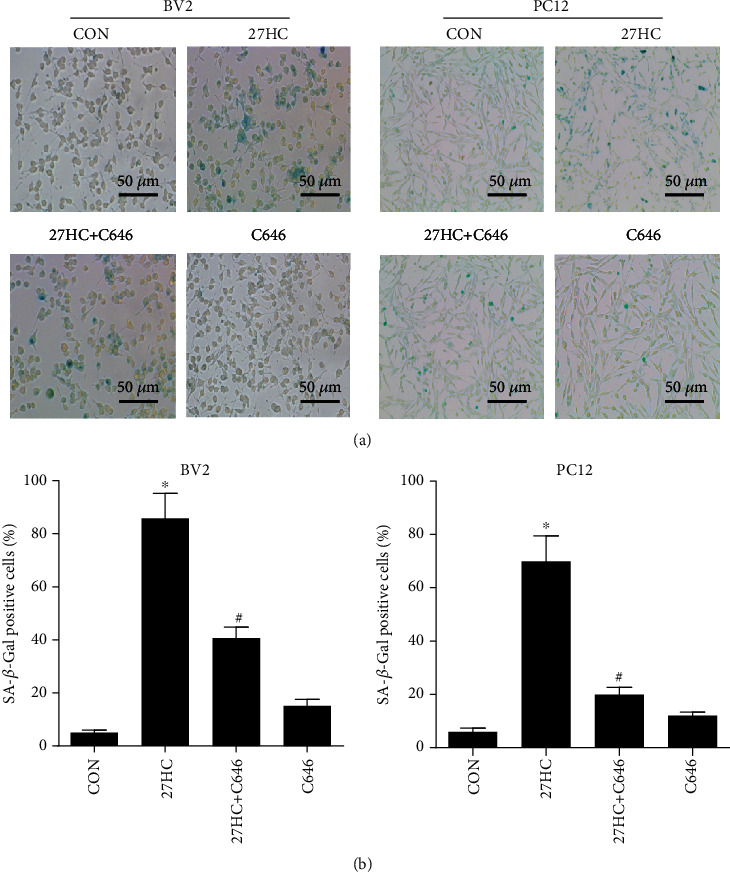
Effects of acetylation on 27HC-induced senescence in neural cells. BV2 and PC12 cells were pretreated with 10 *μ*M 27HC or 10 *μ*M 27HC plus 10 *μ*M C646 for 12 h. CON group: ethanol and DMSO treatment. (a) Cells were subjected to an SA-*β*-Gal staining assay. Scale bars: 50 *μ*m. (b) The aged cells were stained in blue, and the stained cells from panels of BV2 and PC12 cells were counted. ^∗^*P* < 0.05, statistically significant difference from the CON group (BV2: *P* < 0.0001; PC12: *P* < 0.0001). ^#^*P* < 0.05, statistically significant difference from the 27HC-treated group (BV2: *P* = 0.0001; PC12: *P* < 0.0001). Data are expressed as the mean ± SD of three independent experiments.

Our previous work showed that 27HC can induce STAT3 phosphorylation at Tyr705, and this was involved in nerve cell senescence [5]. Both acetylation and phosphorylation of STAT3 play important roles in transcriptional regulation. In the present research, we investigated whether STAT3 acetylation is also involved in 27HC-induced neural cell senescence. BV2 and PC12 cells were treated with 10 *μ*M 27HC, 10 *μ*M 27HC + 10 *μ*M C646, or 10 *μ*M C646 alone for 12 h and then subjected to an SA-*β*-Gal staining assay. We found that 27HC increased the number of SA-*β*-Gal-positive cells. C646, a selective inhibitor of the acetyltransferase P300 associated with STAT3 acetylation, significantly reduced the number of SA-*β*-Gal-positive cells increased by 27HC (Figures 1(a) and 1(b)), indicating that acetylation may be involved in 27HC-induced senescence in neural cells.

### 3.2. 27HC Induces STAT3 Activation via SIRT1

**Figure 2:**
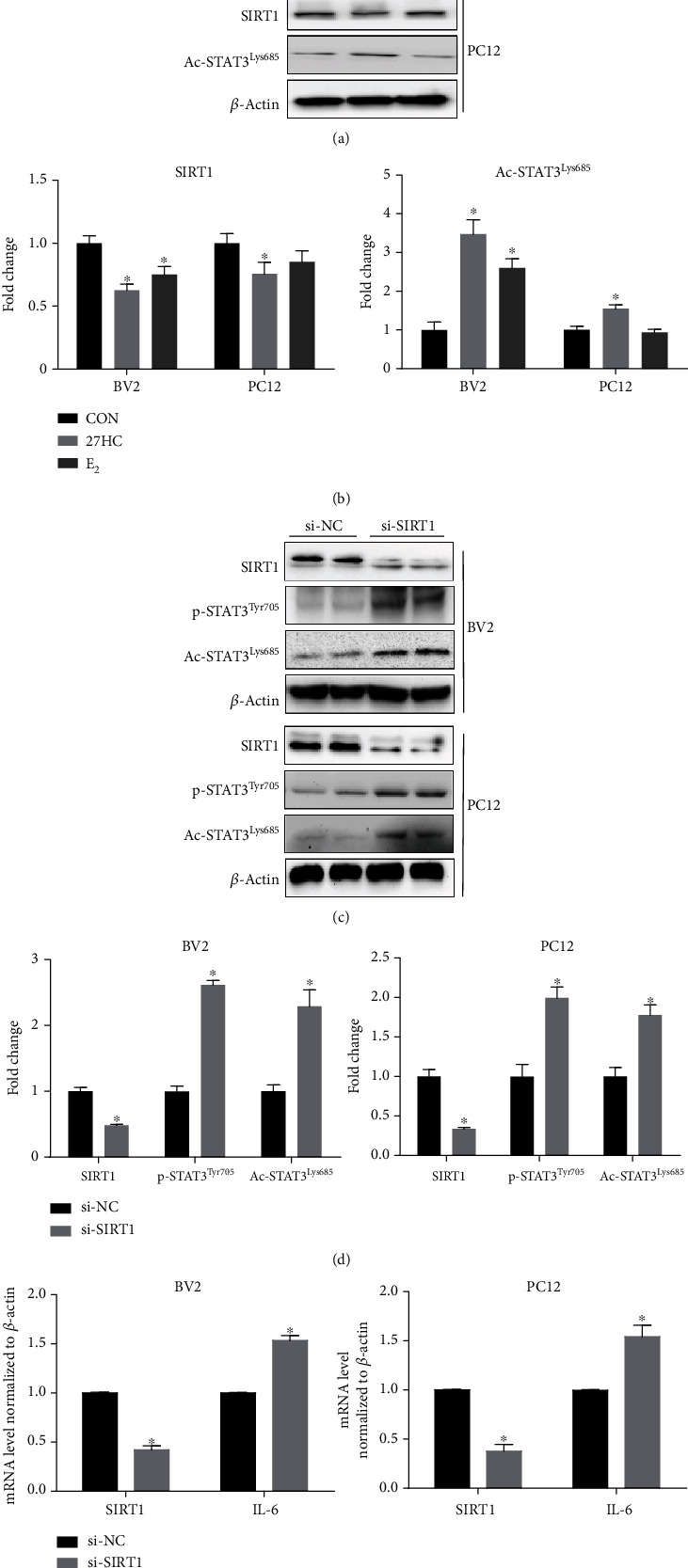
The 27HC induces STAT3 activation via SIRT1. (a) BV2 and PC12 cells were treated with 10 *μ*M 27HC and 10 nM E2. Western blot analysis was performed for SIRT1, Ac-STAT3Lys685, and *β*-Actin expression. CON group: ethanol treatment. (b) The quantitative analysis of protein bands of (a). ^∗^*P* < 0.05, statistically significant difference from the CON group. SIRT1: (BV2: *P* = 0.0011, 27HC group; *P* = 0.0082, E_2_ group) (PC12: *P* = 0.0248, 27HC group; *P* = 0.0947, E2 group); AC-STAT3^Lys685^: (BV2: *P* = 0.0005, 27HC group; *P* = 0.0009, E_2_ group) (PC12: *P* = 0.0026, 27HC group; *P* = 0.4597, E_2_ group). Data are expressed as the mean ± SD of three independent experiments. (c) BV2 and PC12 cells were treated with si-NC or siRNA-SIRT1 for 12 h. Western blot analysis was performed for SIRT1, p-STAT3^Tyr705^, Ac-STAT3^Lys685^, and *β*-actin expression. The two sets are experimental replicates. (d) The quantitative analysis of protein bands of (c). ^∗^*P* < 0.05, statistically significant difference from the si-NC group. BV2: *P* < 0.0001, SIRT1; *P* < 0.0001, p-STAT3^Tyr705^; *P* < 0.0001, Ac-STAT3^Lsy685^; PC12: *P* < 0.0001, SIRT1; *P* = 0.0003, p-STAT3^Tyr705^; *P* = 0.0001, Ac-STAT3^Lsy685^. (e) qRT-PCR analysis of IL-6 and SIRT1. ^∗^*P* < 0.05, statistically significant difference from the si-NC group. (BV2: *P* = 0.0027, SIRT1; *P* = 0.0044, IL-6; PC12: *P* < 0.0001, SIRT1; *P* = 0.0013, IL-6). Data are expressed as the mean ± SD of three independent experiments.

SIRT1 is an NAD-dependent deacetylase that is located in the nucleus. We explored whether SIRT1 can deacetylate STAT3 at Lys685. We initially treated BV2 and PC12 cells with 10 *μ*M 27HC or 10 nM E_2_. We observed that 27HC and E_2_ increased the expression of Ac-STAT3^Lys685^ but decreased the expression of SIRT1 in BV2 cells. 27HC also increased the expression of Ac-STAT3^Lys685^ and decreased the expression of SIRT1 in PC12 cells, but no significant difference was observed in the effects of E2 on the two proteins compared with CON (Figures 2(a) and 2(b)To investigate the role of SIRT1 in STAT3 phosphorylation and acetylation, siRNA-SIRT1 was used to knockdown the expression of SIRT1 in BV2 and PC12 cells. We found that siRNA-SIRT1 increased the levels of p-STAT3^Tyr705^ and Ac-STAT3^Lys685^ in both BV2 and PC12 cells (Figures 2(c) and 2(d)) and also increased the mRNA expression of interleukin 6 (IL-6), a gene related to STAT3 signal activation (Figure 2(e)). Taken together, these data indicated that STAT3 acetylation at Lys685 played a crucial role in 27HC-triggered cellular senescence in neural cells and SIRT1 was involved in 27HC-induced STAT3 activation.

### 3.3. 27HC Downregulates SIRT1 Expression by ROS-Mediated Methylation

**Figure 3:**
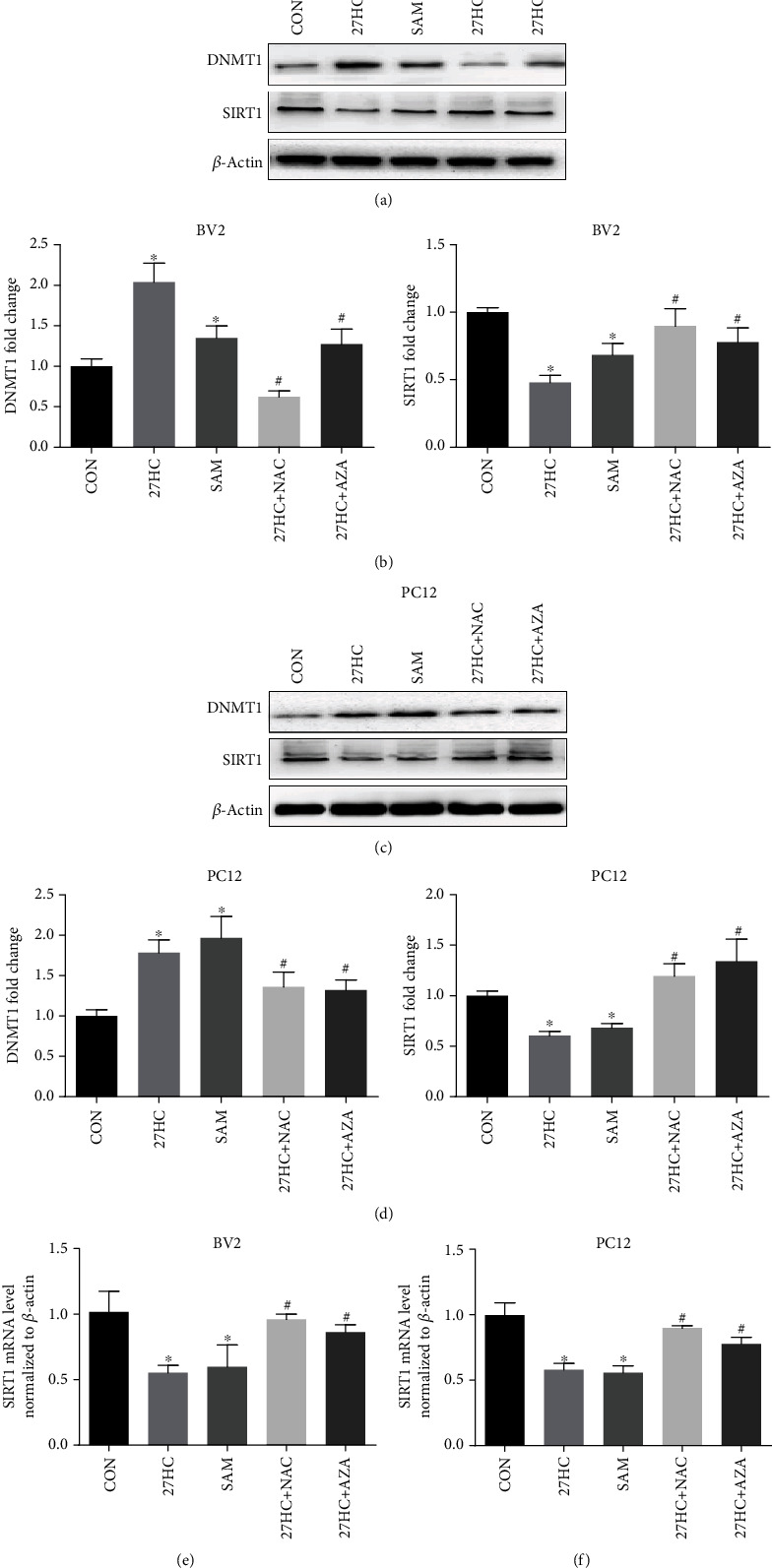
The 27HC downregulates SIRT1 expression via ROS-mediated methylation. BV2 and PC12 cells were treated with 10 *μ*M 27HC, 100 *μ*M SAM, 10 *μ*M 27HC + 2 mM NAC (pretreatment for 2 h), and 10 *μ*M 27HC + 2.5 *μ*M AZA (pretreatment for 2 h) for 12 h; CON group: ethanol treatment. (a, c) Western blot analysis of DNMT1, SIRT1, and *β*-actin. (b, d) The quantitative analysis of protein bands of (a, c). ∗*P* < 0.05, statistically significant difference from the CON group. (b) BV2: (DNMT1: *P* = 0.0020, 27HC group; *P* = 0.0246, SAM group; SIRT1: *P* = 0.0001, 27HC group; *P* = 0.0039, SAM group). (d) PC12: (DNMT1: *P* = 0.0015, 27HC group; *P* = 0.0036, SAM group; SIRT1: *P* = 0.0004, 27HC group; *P* = 0.0009, SAM group). ^#^*P* < 0.05, statistically significant difference from the 27HC-treated group. (b) BV2: (DNMT1: *P* = 0.0006, 27HC+NAC group; *P* = 0.0116, 27HC+AZA group; SIRT1: *P* = 0.0061, 27HC+NAC group; *P* = 0.0124, 27HC+AZA group). (d) PC12: (DNMT1: *P* = 0.0387, 27HC+NAC group; *P* = 0.0166, 27HC+AZA group; SIRT1: *P* = 0.0013, 27HC+NAC group; *P* = 0.0048, 27HC+AZA group). (e, f) qRT-PCR analysis of SIRT1. ^∗^*P* < 0.05, statistically significant difference from the CON group. (e) BV2: *P* = 0.0014, 27HC group; *P* = 0.0104, SAM group. (f) PC12: *P* = 0.0002, 27HC group; *P* = 0.0002, SAM group. ^#^*P* < 0.05, statistically significant difference from the 27HC-treated group. (e) BV2: *P* < 0.0001, 27HC+NAC group; *P* = 0.0003, 27HC+AZA group. (f) PC12: *P* < 0.0001, 27HC+NAC group; *P* = 0.0014, 27HC+AZA group. Data are expressed as the mean ± SD of three independent experiments.

As described above, our data showed that 27HC can inhibit SIRT1 and increased Ac-STAT3^Lys685^. Studies have shown that ROS induces cell DNA damage as well as DNA methylation leading to the silencing of some genes [28]. In the present study, we explored whether the regulation of SIRT1 genes by 27HC was associated with epigenetic changes. BV2 and PC12 cells were exposed to 10 *μ*M 27HC, 100 *μ*M SAM, 10 *μ*M 27HC + 2 mM NAC, and 10 *μ*M 27HC + 2.5 *μ*M AZA, respectively. We found that 27HC or SAM (methyl-donor) alone promoted the expression of the DNA methyltransferase DNMT1 and inhibited the expression of SIRT1. Simultaneously, the addition of the methyltransferase inhibitor AZA reversed the upregulation of DNMT1 and inhibition of SIRT1 induced by 27HC (Figures 3(a)–3(d)). Moreover, we also observed that the addition of the ROS scavenger NAC reversed the inhibitory effect of 27HC on SIRT1 at both an mRNA (Figures 3(e) and 3(f)) and protein level (Figures 3(a)–3(d)). These findings suggested that 27HC downregulated SIRT1 expression through DNA methylation mediated by ROS.

### 3.4. REV Alleviates 27HC-Induced Senescence and STAT3 Activation

**Figure 4:**
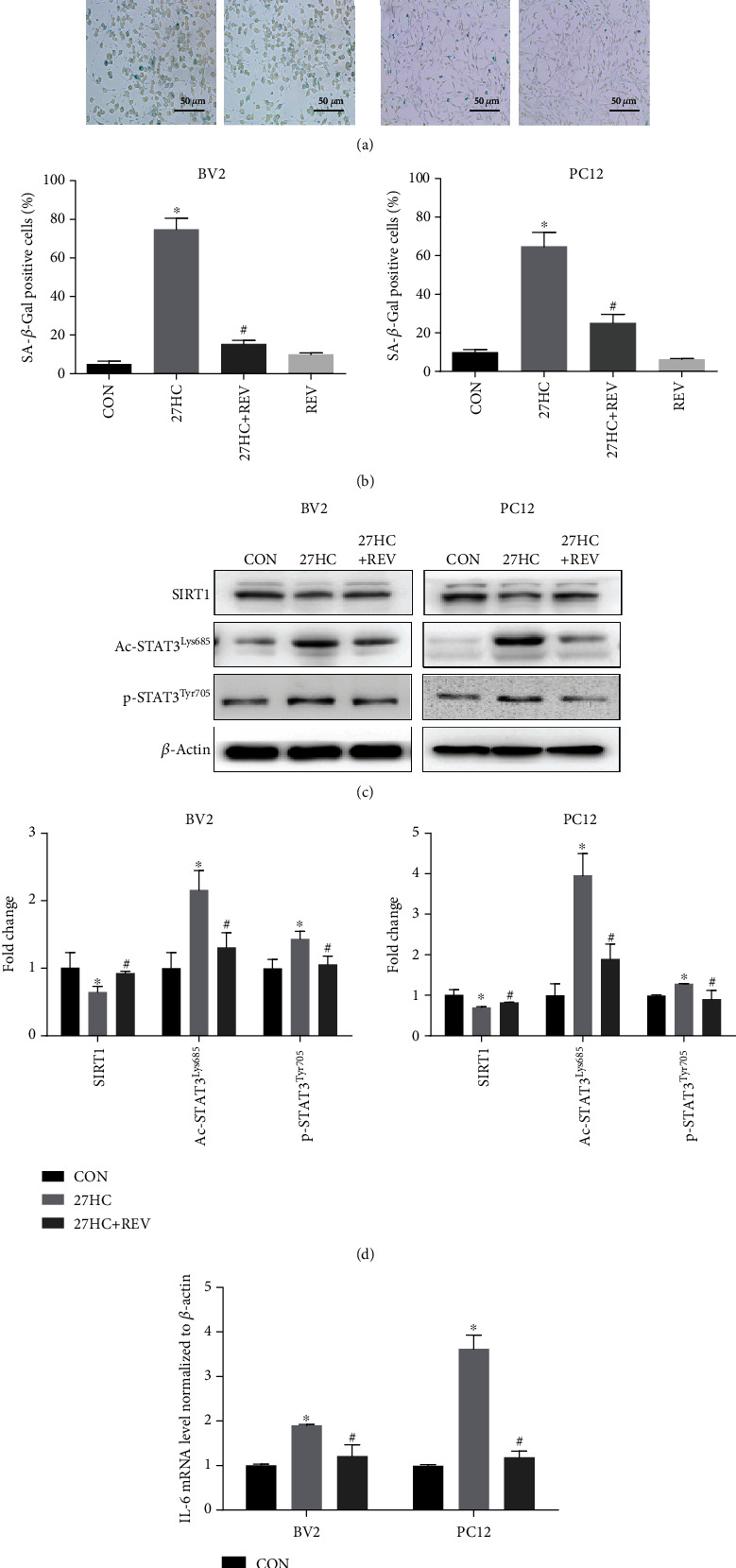
REV inhibits 27HC-induced senescence and STAT3 activation. BV2 and PC12 cells were treated with 10 *μ*M 27HC, 10 *μ*M 27HC + 20 *μ*M REV, or 20 *μ*M REV alone for 12 h. CON group: ethanol treatment. (a) Cells were subjected to an SA-*β*-Gal staining assay. Scale bars: 50 *μ*m. (b) The aged cells were stained in blue, and the number of stained cells from panels was counted. ^∗^*P* < 0.05, statistically significant difference from the CON group. BV2: *P* < 0.0001; PC12: *P* < 0.0001. ^#^*P* < 0.05, statistically significant difference from the 27HC-treated group. BV2: *P* < 0.0001; PC12: *P* < 0.0001. Data are expressed as the mean ± SD of three independent experiments. BV2 and PC12 cells were treated with 10 *μ*M 27HC or 10 *μ*M 27HC + 20 *μ*M REV for 12 h. CON group: ethanol treatment. (c) Western blot analysis of SIRT1, Ac-STAT3^Lys685^, p-STAT3^Tyr705^, and *β*-actin. (d) The quantitative analysis of protein bands of (c). ^∗^*P* < 0.05, statistically significant difference from the CON group. BV2: (SIRT1: *P* = 0.0224; Ac-STAT3^Lys685^: *P* = 0.0053; p-STAT3^Tyr705^: *P* = 0.0129); PC12: (SIRT1: *P* = 0.0155; Ac-STAT3^Lys685^: *P* = 0.0011; p-STAT3^Tyr705^: *P* < 0.0001). ^#^*P* < 0.05, statistically significant difference from the 27HC-treated group. BV2: (SIRT1: *P* = 0.0007; Ac-STAT3^Lys685^: *P* = 0.0144; p-STAT3^Tyr705^: *P* = 0.0176); PC12: (SIRT1: *P* < 0.0001; Ac-STAT3^Lys685^: *P* = 0.0053; p-STAT3^Tyr705^: *P* = 0.0368). (e) qRT-PCR analysis of IL-6. ^∗^*P* < 0.05, statistically significant difference from the CON group. BV2: *P* = 0.0006; PC12: *P* < 0.0001. ^#^*P* < 0.05, statistically significant difference from the 27HC-treated group. BV2: *P* = 0.0023; PC12: *P* < 0.0001. Data are expressed as the mean ± SD of three independent experiments.

To confirm whether the antioxidant properties of REV were related to 27HC-induced cellular senescence, we treated BV2 and PC12 cells with 10 *μ*M 27HC, 10 *μ*M 27HC + 20 *μ*M REV, or 20 *μ*M REV alone for 12 h. Interestingly, REV significantly attenuated the 27HC-enhanced number of SA-*β*-Gal-positive cells in both cell types (Figures 4(a) and 4(b)). Both acetylation and phosphorylation of STAT3 play important roles in transcriptional regulation. So, we investigated the effect of REV on STAT3 in neural cells. BV2 and PC12 cells were exposed to 10 *μ*M 27HC or 10 *μ*M 27HC + 20 *μ*M REV for 12 h. We found that REV weakened the suppressive function of 27HC on SIRT1 and inhibited the expression of p-STAT3^Tyr705^ and Ac-STAT3^Lys685^ induced by 27HC (Figures 4(c) and 4(d)). In addition, REV also inhibited the 27HC-induced increase in IL-6 mRNA (Figure 4(e)). The above results indicated that REV attenuated 27HC-enhanced cellular senescence and inhibited the activation of STAT3 induced by 27HC.

### 3.5. Effects of 27HC on ROS Generation in Zebrafish Larva

**Figure 5:**
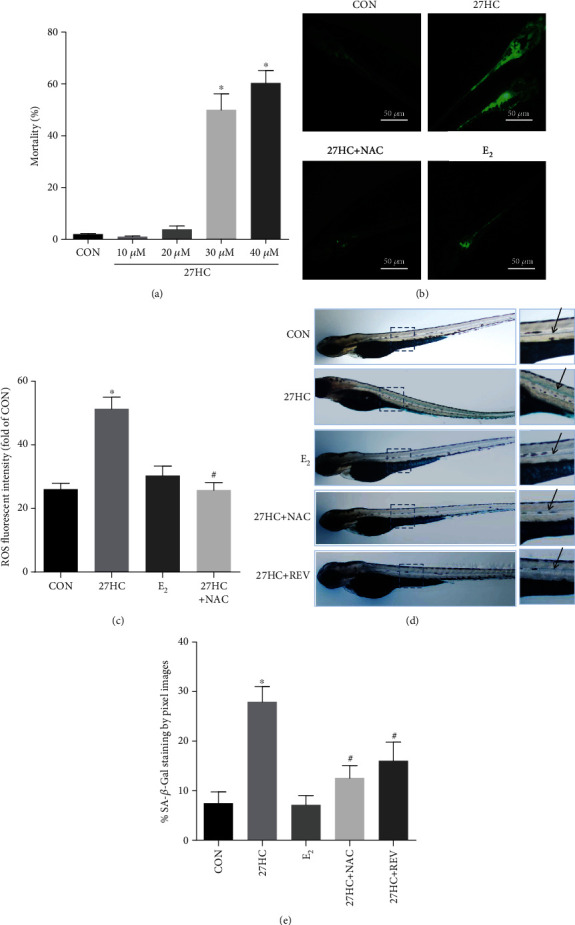
Effects of 27HC on ROS generation in zebrafish larvae. (a) Effect of different concentrations of 27HC on zebrafish embryo mortality (*n* = 50). CON group: ethanol treatment. ^∗^*P* < 0.05, statistically significant difference from the CON group. *P* = 0.9927, 10 *μ*M 27HC group; *P* = 0.9071, 20 *μ*M 27HC group; *P* < 0.0001, 30 *μ*M 27HC group; *P* < 0.0001, 40 *μ*M 27HC group. (b) Staining for ROS in larvae at 4 dpf following 36 hpf to 4 dpf treatment with 20 *μ*M 27HC, 20 *μ*M 27HC + 20 *μ*M NAC, or 10 nM E2. CON group: ethanol treatment. Scale bars: 50 *μ*m. (c) The levels of ROS fluorescence were determined using flow cytometric analysis (*n* = 30). REV attenuated 27HC-induced aging in zebrafish larvae. ^∗^*P* < 0.05, statistically significant difference from the CON group (*P* < 0.0001). ^#^*P* < 0.05, statistically significant difference from the 27HC-treated group (*P* < 0.0001). (d) SA-*β*-Gal staining in zebrafish embryos at 4 dpf following 36 hpf to 4 dpf treatment with 20 *μ*M 27HC, 10 nM E2, 20 *μ*M 27HC + 20 *μ*M NAC, or 20 *μ*M 27HC + 1 *μ*M REV. CON group: ethanol treatment. (e) Quantitative analysis of SA-*β*-Gal staining in larvae (*n* = 30). ^∗^*P* < 0.05, statistically significant difference from the CON group (*P* < 0.0001). ^#^*P* < 0.05, statistically significant difference from the 27HC-treated group. *P* = 0.0002, 27HC+NAC group; *P* = 0.0013, 27HC+REV group. Data are expressed as mean ± SD of three independent experiments.

The zebrafish is inexpensive to maintain and has favorable characteristics for experimentation, such as a high fecundity, rapid external development, embryonic translucence, and ease of genetic manipulation. The senescence process in zebrafish shares similarities with humans and mammals to a certain extent, and zebrafish have been gradually used in the study of aging biology in recent years [29]. Therefore, we used zebrafish as an in vivo experimental model to further verify the effect of 27HC on neurobehavior and aging. We first observed zebrafish embryo mortality after exposure to 0, 10, 30, and 40 *μ*M 27HC from 36 hpf to 4 dpf. The results showed that zebrafish embryo mortality was increased in a dose-dependent manner (Figure 5(a)). Then, we selected 20 *μ*M 27HC (zebrafish embryo mortality < 5%) as the experimental dose. Zebrafish larvae were treated with 20 *μ*M 27HC, 10 nM E_2_, and 20 *μ*M 27HC + 20 *μ*M NAC for 36 hpf to 4 dpf. In vivo staining for ROS at 4 dpf with DCFH-DA revealed the presence of high levels of ROS in zebrafish larvae exposed to 27HC, which was insignificant in 10 nM E_2_-exposed larvae. The effect of 27HC on ROS generation in zebrafish larvae was also inhibited following combination with NAC (Figures 5(b) and 5(c)).

### 3.6. REV Attenuated 27HC-Induced Aging in Zebrafish Larvae

The zebrafish embryos were exposed to 20 *μ*M 27HC, 10 nM E_2_, 20 *μ*M 27HC + 20 *μ*M NAC, or 20 *μ*M 27HC + 1 *μ*M REV for 36 hpf to 4 dpf. Staining for SA-*β*-Gal in the neural spinal cord was found to increase in the zebrafish embryos following 27HC exposure, yet the staining pattern was distinct from E_2_. Further, we found that 20 *μ*M NAC or 1 *μ*M REV could reduce the 27HC-induced SA-*β*-Gal activity (Figures 5(d) and 5(e)). These results showed that REV improved the 27HC-induced aging in the neural spinal cord of zebrafish larvae.

### 3.7. Effects of REV on 27HC-Induced Disorders in Locomotor Behavior of Zebrafish Larvae

**Figure 6:**
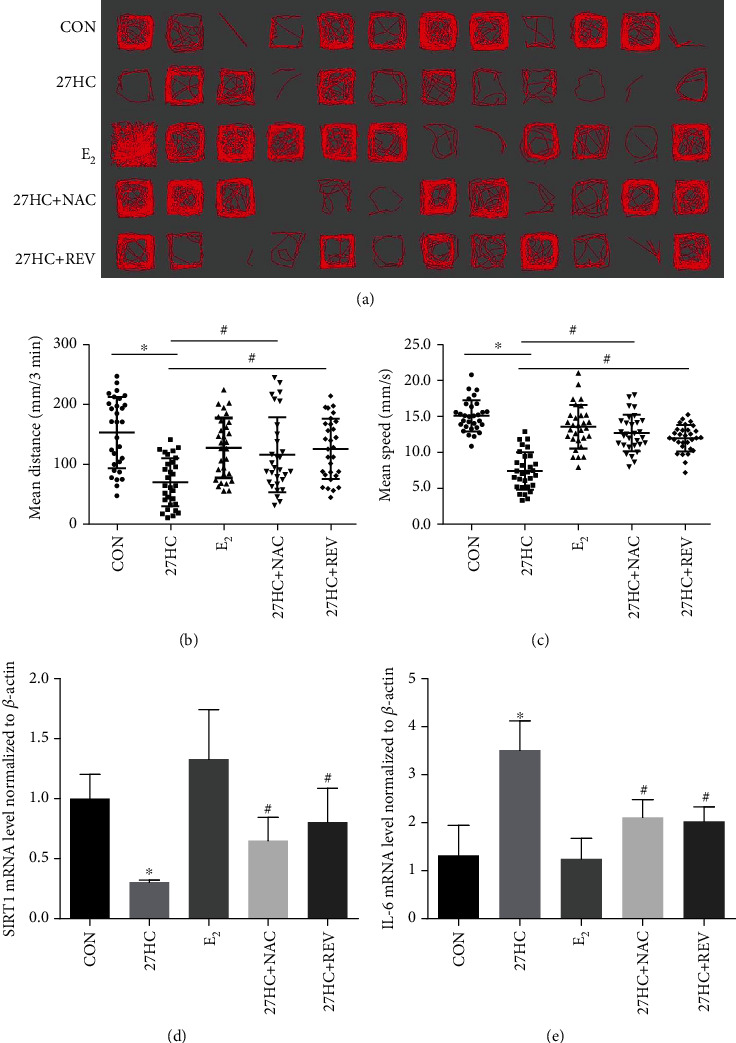
Effects of REV on 27HC-induced locomotor disorders in zebrafish larvae. Zebrafish larvae from 36 hpf to 4 dpf were exposed to 20 *μ*M 27HC, 10 nM E2, 20 *μ*M 27HC + 20 *μ*M NAC, or 20 *μ*M 27HC + 1 *μ*M REV. CON group: ethanol treatment. (a) Locomotor behavior of zebrafish larvae was observed using EthoVision XT software Noldus IT. (b, c) Analysis of behavioral parameters of zebrafish larvae (*n* = 30). ^∗^*P* < 0.05, statistically significant difference from the CON group. (b) *P* < 0.0001. (c) *P* < 0.0001. ^#^*P* < 0.05, statistically significant difference from the 27HC-treated group. (b) *P* = 0.0013, 27HC+NAC group; *P* < 0.0001, 27HC+REV group. (c) *P* < 0.0001, 27HC+NAC group; *P* < 0.0001, 27HC+REV group. Data are expressed as the mean ± SD of three independent experiments. (d, e) qRT-PCR analysis of SIRT1 and IL-6. ^∗^*P* < 0.05, statistically significant difference from the CON group (*n* = 30). (d) *P* = 0.0248. (e) *P* = 0.0123. ^#^*P* < 0.05, statistically significant difference from the 27HC-treated group. (d) *P* = 0.0368, 27HC+NAC group; *P* = 0.378, 27HC+REV group. (e) *P* = 0.0276, 27HC+NAC group; *P* = 0.0198, 27HC+REV group. Data are expressed as the mean ± SD of three independent experiments.

The effects of 27HC on locomotor behavior and behavioral parameters of zebrafish larva were evaluated by EthoVision XT software Noldus IT after a 36 hpf to 4 dpf exposure to 20 *μ*M 27HC, 10 nM E_2_, 20 *μ*M 27HC + 20 *μ*M NAC, or 20 *μ*M 27HC + 1 *μ*M REV. We visualized the locomotor trajectories of the 4 dpf larvae in the dark for 3 min. The results showed that 70% of the larvae in the 27HC-exposed group no longer turned routinely along the inner wall of the 96-well plate compared to the control group, which was accompanied by an increase in spontaneous locomotor trajectories, stereotyped steering, small-scale circle phenomena, and touch-wall reflex activity reduction. At the same time, this irregular movement of larvae was alleviated by combining 27HC with NAC or REV (Figure 6(a)).

In addition, the analysis of behavioral parameters revealed that, compared with the control group, the larvae moved a shorter distance and had a slower mean speed after 27HC exposure. The inhibitory effects of 27HC on the spontaneous movement distance and mean speed of the larvae were weakened following combination with NAC or REV (Figures 6(b) and 6(c)).

The mRNA expression of SIRT1 and IL-6 in each group was detected by qRT-PCR. The results showed that SIRT1 expression was significantly inhibited after exposure to 27HC, whereas IL-6 expression was increased. After combining 27HC with NAC or REV, the effects of 27HC on SIRT1 and IL-6 were reversed (Figures 6(d) and 6(e)). These results were consistent with our in vitro experiments. In the E_2_-treated group, there was no detectable change in larvae (Figures 6(a)–6(e)). All of the above results showed that 27HC induced locomotor behavior disorders and inflammatory cytokines in zebrafish larvae, and REV alleviated 27HC-induced aging and locomotor behavior disorders.

## 4. Discussion

Animal studies have shown that the passage of serum 27HC through the BBB can result in the deposition of oxidized cholesterol in the brain, resulting in metabolic disorders of *β*-amyloid (A*β*) and impaired spatial learning and memory [30]. Another study has suggested that high levels of circulating cholesterol increase the entry of 27HC into the brain, which may induce learning and memory impairment [31]. Based on this evidence, 27HC may be associated with neurodegenerative processes in the brain. 27HC may negatively modulate cognitive effects and interrupt neuronal cells and thereby lead to neurodegenerative diseases, such as aging or Alzheimer's disease (AD).

More and more studies have confirmed that SIRT1 plays an important role in the development of neurodegenerative diseases, such as AD, Parkinson's disease (PD), or ischemic stroke [32–34]. It is well-known that the activity of SIRT1 is affected by NAD+, which is an important enzyme substrate for SIRT1. NAD+ is also an electron transporter in the mitochondrial respiratory chain and is affected by the redox state. Therefore, it is believed that the in vivo environment, oxidative stress, and metabolic changes can all affect the acetylation enzyme activity of SIRT1 [35]. More evidence showed that elevated ROS levels can directly or indirectly control SIRT1 enzyme activity. For example, with aging and some age-related diseases, the overproduction of ROS weakens the function of SIRT1 [36]. In addition, ROS can inhibit SIRT1 mRNA levels by inducing microRNA expression [37].

In the present study, we explored whether the regulation of SIRT1 genes by 27HC was associated with epigenetic changes. We used the methyl-donor SAM and the methyltransferase inhibitor AZA. And the results suggested that 27HC and SAM could promote the DNMT1 expression and inhibit the SIRT1 expression, and the addition of the methyltransferase inhibitor AZA reversed the upregulation of DNMT1 and inhibition of SIRT1 induced by 27HC. Furthermore, the ROS scavenger NAC reversed the effect of 27HC on DNMT1 and SIRT1. Based on the above results, we considered that 27HC could induce SIRT1 DNA methylation by promoting methyltransferase DNMT1 expression. NNMT may regulate not only SIRT1 gene; whether there are aging-related genes regulated by NNMT in the process of 27HC induced-aging remains to be further explored. We found that the mRNA level and protein level showed consistent changes, and there is no evidence that SIRT1 protein degradation was involved in the process of 27HC-induced cellular senescence, which needs to be further explored. We examined the effects of 27HC and SAM on DNMT1. SAM is a donor which provides the methyl groups for histone or nucleic acid modification. DNMT1 is a key enzyme for DNA replication and repair and maintenance of methylation level [38]. So we concluded that the downregulation of SIRT1 was related to upregulation of DNMT1 induced by 27HC. The SAM treatment indirectly shows the expression of DNMT1 and its effect on SIRT1 expression silence, in which as a limitation in the present study, a more conclusive experiment would be to perform bisulfite conversion sequencing or digestion-based PCR assay to demonstrate the DNA methylation site of SIRT1 gene changed by 27HC in the process of cellular senescence.

The phytochemical REV is a classical SIRT1 agonist, and the covalent binding of REV to SIRT1 alters the molecular conformation of SIRT1 and increases the affinity of the protein for its substrate [39, 40]. Our study also confirmed that REV can alleviate 27HC-enhanced cellular senescence and the inhibition of SIRT1 induced by 27HC in neural cells. Our in vivo study further indicated that REV alleviated 27HC-induced spinal cord senescence and locomotor disorders in zebrafish larvae. Extensive research has shown the interventional effects of REV in vitro and vivo, and the form of REV used mostly is prototype rather than its metabolites. In the present study, we also observed that REV prototype alleviated 27HC-induced aging and locomotor behavior disorders in zebrafish. Whether the intervention of REV is its prototype or its metabolites is unclear in this study. Resveratrol has a high metabolism, leading to the production of conjugated sulfates, glucuronides, which retain some biological activity [41]. The fact that resveratrol metabolite was less tested also makes these results poorly translatable. Here, which metabolites of resveratrol are involved in the intervention has not been explored. So, when these questions are clearly identified, we will further explore the intervention functions of resveratrol and its metabolites at physiological concentrations.

Changes in the levels of STAT3 phosphorylation and acetylation were observed in A*β*-induced microglial cell activation models [42]. Therefore, activation of STAT3 also plays an important role in nerve injury and neurodegenerative diseases. Moreover, it is worth noting that the acetylation site of STAT3 is very important for the stability of its dimer formation and gene transcription. Yuan and his colleagues first reported the acetylation of STAT3 at the lysine 685 (Lys685) site, which in turn regulates its dimer formation and DNA-binding capacity [13]. In our previous study, we used a Stattic (10 *μ*M) to inhibit the level of p-STAT3, which further inhibited the mRNA expression and secretion of IL6 promoted by 27HC and 27HC-induced cellular senescence [5], suggesting that STAT3 activation is closely related to 27HC-induced cell senescence. Here, in addition to C646, we also used REV, a naturally occurring phytoalexin that inhibits STAT3 acetylation, and this function is the same with C646 [43]. We found that REV inhibited the expression of p-STAT3^Tyr705^ and Ac-STAT3^Lys685^ promoted by 27HC, which alleviates the 27HC-induced cellular senescence (Figures 4). We considered that STAT3 acetylation may be involved in the cellular senescence induced by 27HC based on our results.

Current studies showed that both STAT3 phosphorylation and STAT3 acetylation play important functions in transcriptional regulation. The role of STAT3 phosphorylation has been well studied, but the research of STAT3 acetylation is relatively new. There are literatures on the regulation between STAT3 phosphorylation and STAT3 acetylation, but it is not clear which is more important. Many studies have suggested that SIRT1 inhibited STAT3 phosphorylation by deacetylating STAT3 [13, 44]. In the present study, we found knockdown SIRT1, the best characterized member of class III HDACs, which increased the expression of the STAT3 Lys685 site, and further upregulated STAT3 phosphorylation in Tyr705 (Figure 2). Furthermore, we found that REV, a naturally occurring phytoalexin that inhibits STAT3 acetylation [43], weakened the suppressive function of 27HC on SIRT1 and then inhibited the expression of Ac-STAT3^Lys685^ and p-STAT3^Tyr705^ promoted by 27HC (Figure 4). Suggesting that STAT3 phosphorylation may regulated by STAT3 acetylation in the cellular senescence induced by 27HC. The mode of regulation between STAT3 phosphorylation and STAT3 acetylation needs to be further clarified.

Here, zebrafish was used as an in vivo model; the results suggested that 27HC promoted the accumulation of ROS and aging in zebrafish larvae, which differed from the protective effects of E_2_. Meanwhile, we found that the ROS scavenger NAC and REV markedly blocked the aging induced by 27HC. Furthermore, our results showed that 70% of the zebrafish larvae in the 27HC-exposed group no longer turned routinely along the inner wall of the 96-well plate compared to the control group, which suggested that 27HC affects spontaneous locomotor behavior. And REV alleviates the effect of 27HC via regulating the level of SIRT1 and IL-6.

## 5. Conclusions

**Figure 7:**
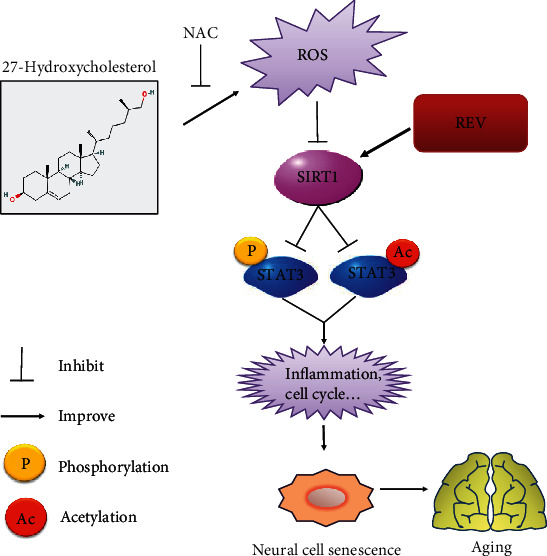
Schematic representation of molecular mechanisms involved in 27HC-induced senescence. 27HC induces a high level of ROS generation, which can downregulate the expression of SIRT1 and subsequently activate the phosphorylation and acetylation of STAT3, which is regulated by SIRT1. Activation of STAT3 triggers aging-related changes in inflammation and the cell cycle leading to neural cell senescence and organism aging.

In summary, our findings demonstrated that 27HC induced neural cell senescence and aging in the neural spinal cord of the zebrafish model. The 27HC induced the acetylation of STAT3 via SIRT1, and the 27HC-induced decrease in SIRT1 was associated with ROS-mediated methylation. Moreover, REV attenuated 27HC-induced senescence by inhibiting STAT3 signaling via SIRT1 in neural cells and zebrafish (Figure 7). In conclusion, REV alleviated 27HC-induced senescence in neural cells and affected zebrafish locomotor behavior by activating SIRT1-mediated STAT3 signaling.

## Figures and Tables

**Table 1:** siRNAs used in this study.

Names	Web link	Source	Used
SIRT1 siRNA (BV2)	https://www.scbt.com/zh/p/sirt1-sirna-m-shrna-and-lentiviral-particle-gene-silencers https://www.scbt.com/zh/p/control-sirna-a	Santa Cruz Biotechnology	25 nM
NC siRNA (BV2)		Santa Cruz Biotechnology	25 nM
SIRT1 siRNA (PC12)	https://http://www.scbt.com/zh/p/sirt1-sirna-r-shrna-and-lentiviral-particle-gene-silencers	Santa Cruz Biotechnology	25 nM
NC siRNA (PC12)	https://http://www.scbt.com/zh/p/control-sirna-a	Santa Cruz Biotechnology	25 nM

**Table 2:** Primer sequence.

Genes	Primers (5′-3′)
RAT-IL-6	Forward: CAGCCAGTTGCCTTCTTG Reverse: TGGTCTGTTGTGGGTGGT
RAT-SIRT1	Forward: GTCTGTGCCTTCCAGTTGCT Reverse: CTGCTTGCTGTCCATACCTG
RAT-*β*-actin	Forward: AAGTCCCTCACCCTCCCAAAAG Reverse: AAGCAATGCTGTCACCTTCCC
Mouse-IL-6	Forward: ACCAGAGGAAATTTTCAATAGGC Reverse: TGATGCACTTGCAGAAAACA
Mouse-SIRT1	Forward: GGAACCTTTGCCTCATCT Reverse: CTGGAACCAACAGCCTTA
Mouse-*β*-actin	Forward: CGATGCCCTGAGGCTCTTT Reverse: TGGATGCCACAGGATTCCAT
Zebrafish-IL-6	Forward: AAGGGGTCAGGATCAGCAC Reverse: GCTGTAGATTCGCGTTAGACATC
Zebrafish-SIRT1	Forward: CAGCTCTGCTACAATTCATCGCGTC Reverse: AATCTCTGTAGATCCAGCGCGTGTG

## Data Availability

The data used to support the findings of the present study are available from the corresponding authors upon request.

## Abbreviations

27HC: 27-Hydroxycholesterol
AZA: Azacitidine
E2: 17*β*-Estradiol
NAC: N-Acetylcysteine
REV: Resveratrol
ROS: Reactive oxygen species
SA-*β*-Gal: Senescence-associated *β*-galactosidase
SAM: S-Adenosyl methionine
SIRT1: Sirtuin-1
STAT3: Signal transducer and activator of transcription 3.
